# Supplementary figures and images for: The seminal odorant binding protein Obp56g is required for mating plug formation and male fertility in Drosophila melanogaster
Source: eLife. 2023 Dec 21;12:e86409. doi: 10.7554/eLife.86409 (PMC10834028; doi:10.7554/eLife.86409)

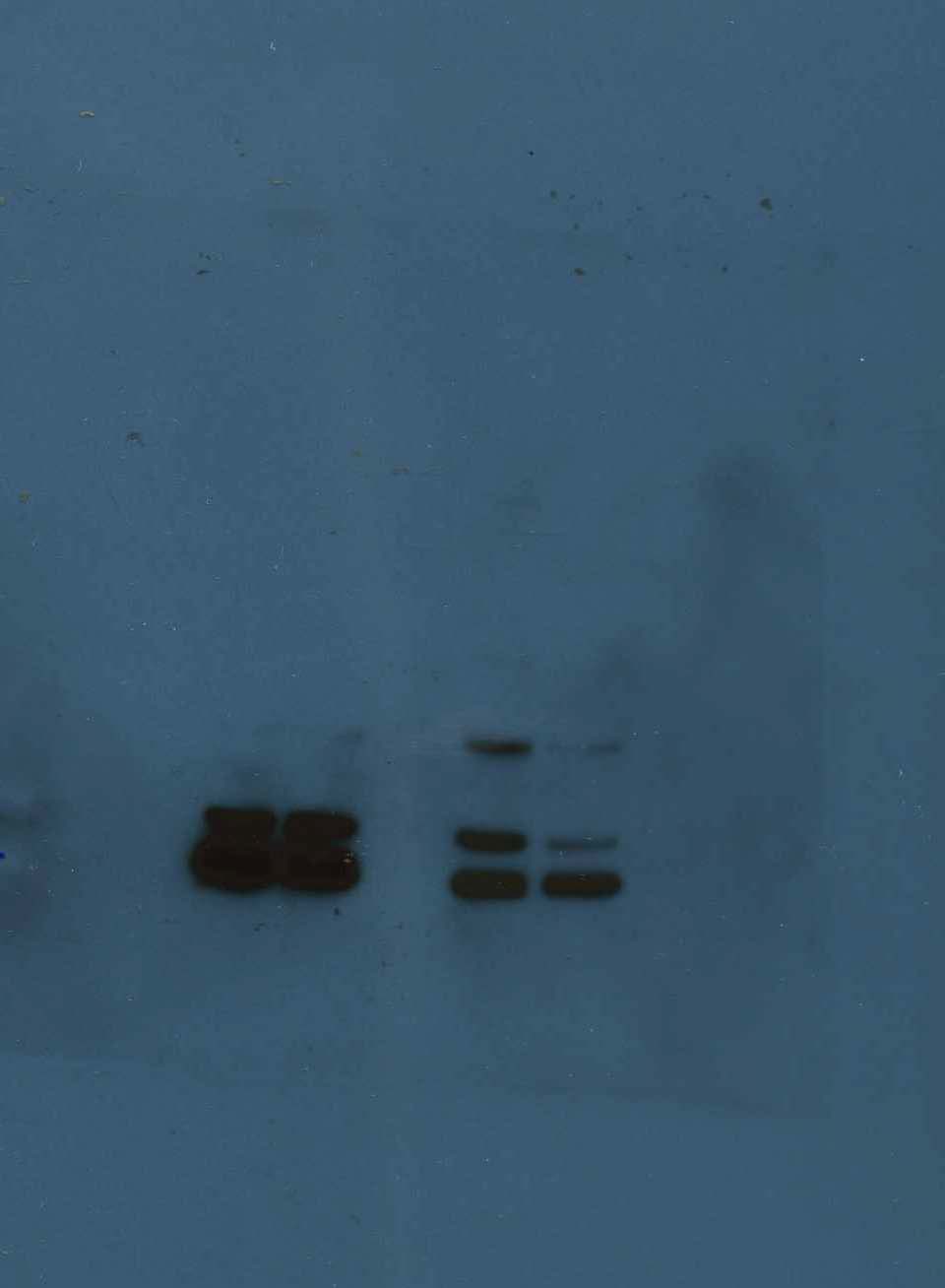

Supplement: Figure 4—figure supplement 2—source data 1. [file elife-86409-fig4-figsupp2-data1.zip › 26Aa_ovulin_raw.jpg]

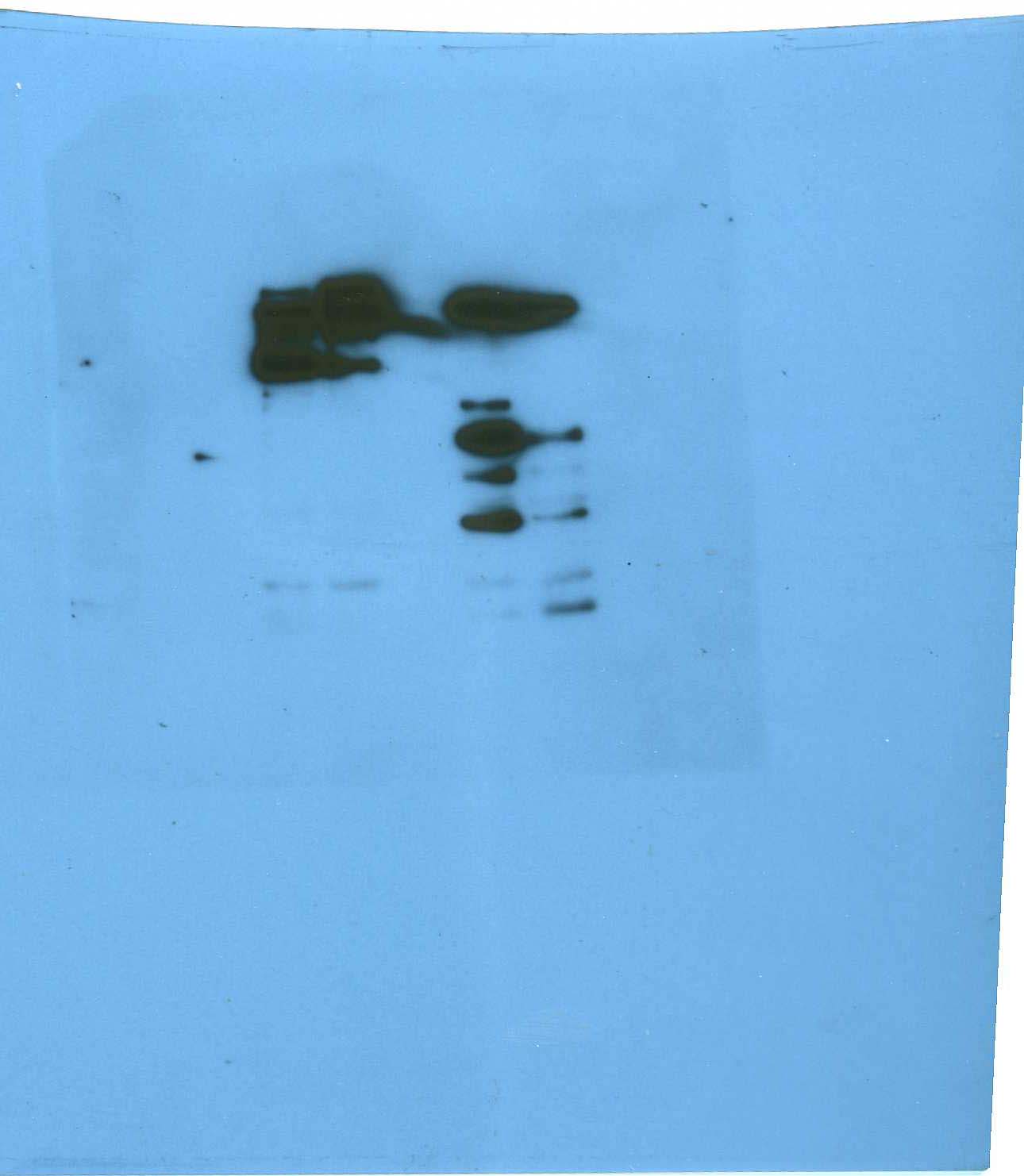

Supplement: Figure 4—figure supplement 2—source data 1. [file elife-86409-fig4-figsupp2-data1.zip › 36DE_raw.jpg]

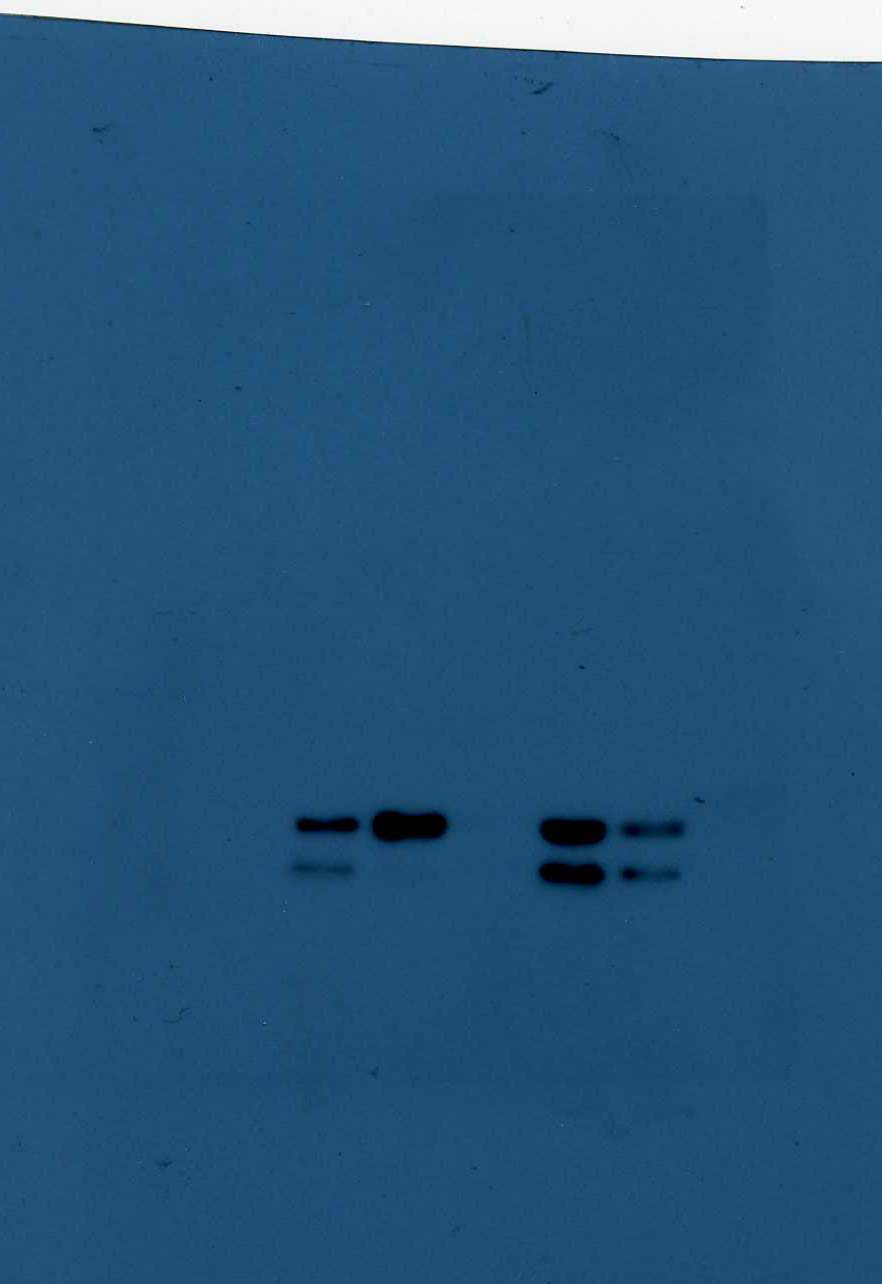

Supplement: Figure 4—figure supplement 2—source data 1. [file elife-86409-fig4-figsupp2-data1.zip › 9997_raw.jpg]

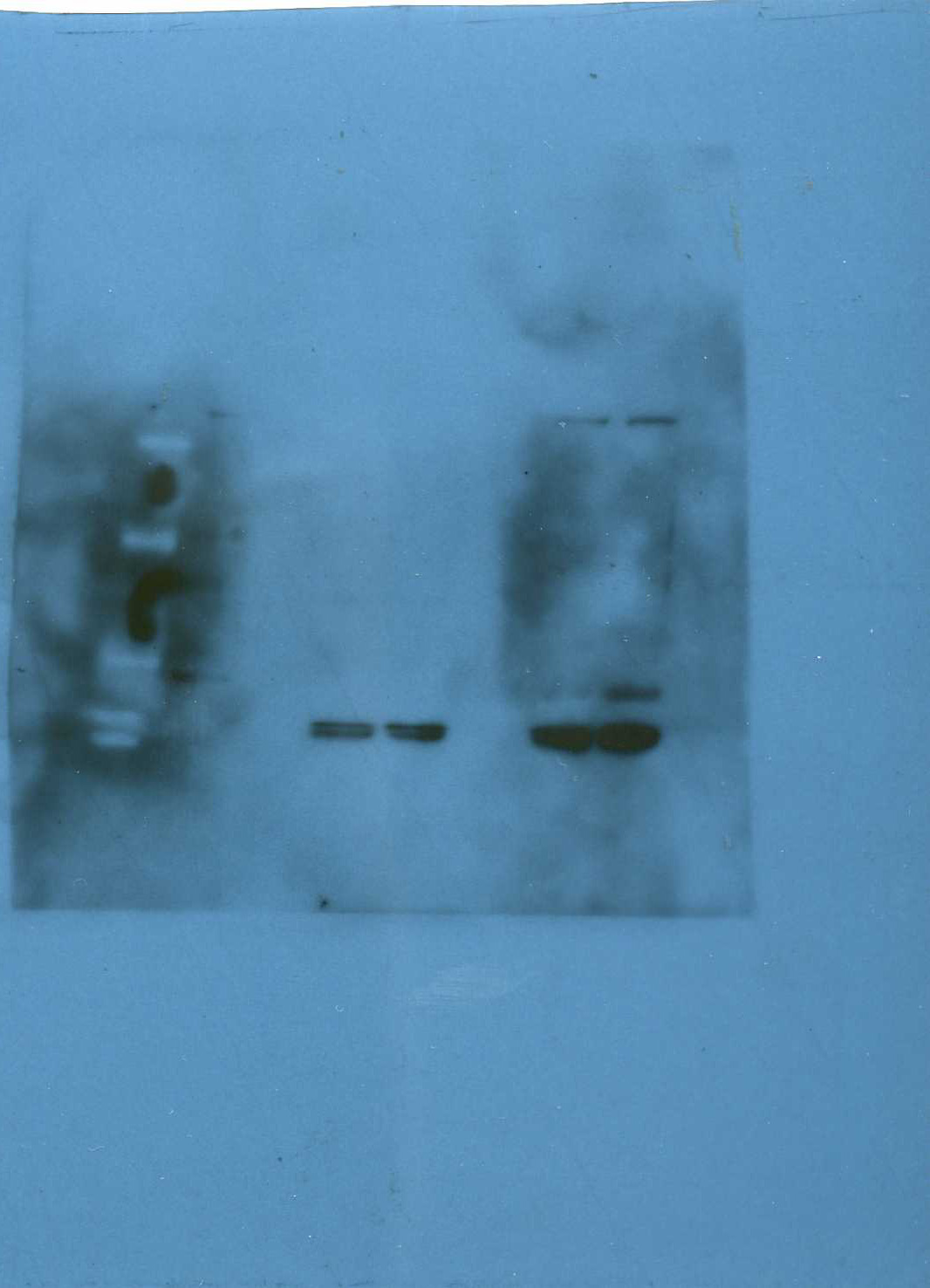

Supplement: Figure 4—figure supplement 2—source data 1. [file elife-86409-fig4-figsupp2-data1.zip › Antr_raw.jpg]

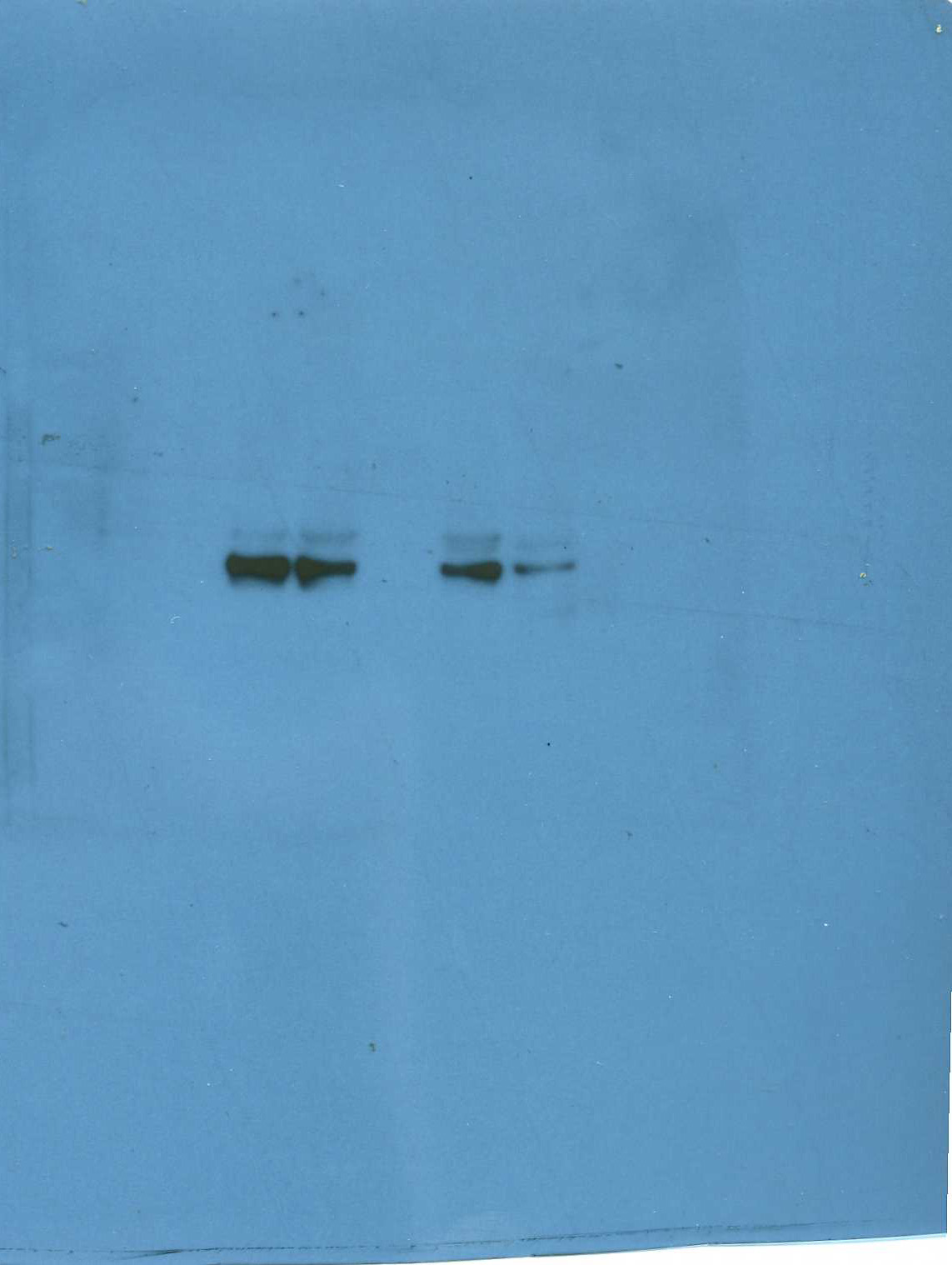

Supplement: Figure 4—figure supplement 2—source data 1. [file elife-86409-fig4-figsupp2-data1.zip › CG1652_raw.jpg]

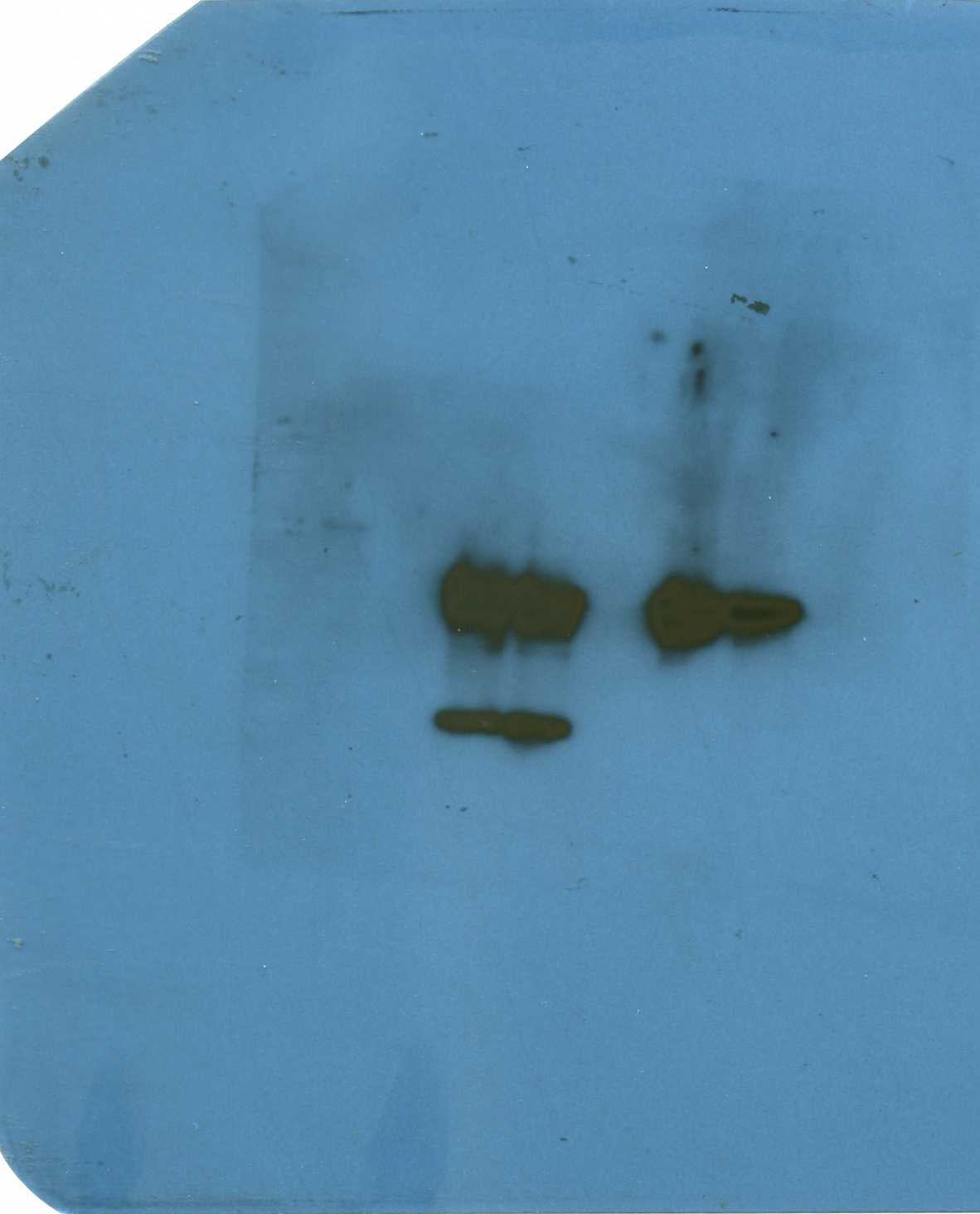

Supplement: Figure 4—figure supplement 2—source data 1. [file elife-86409-fig4-figsupp2-data1.zip › CG1656_raw.jpg]

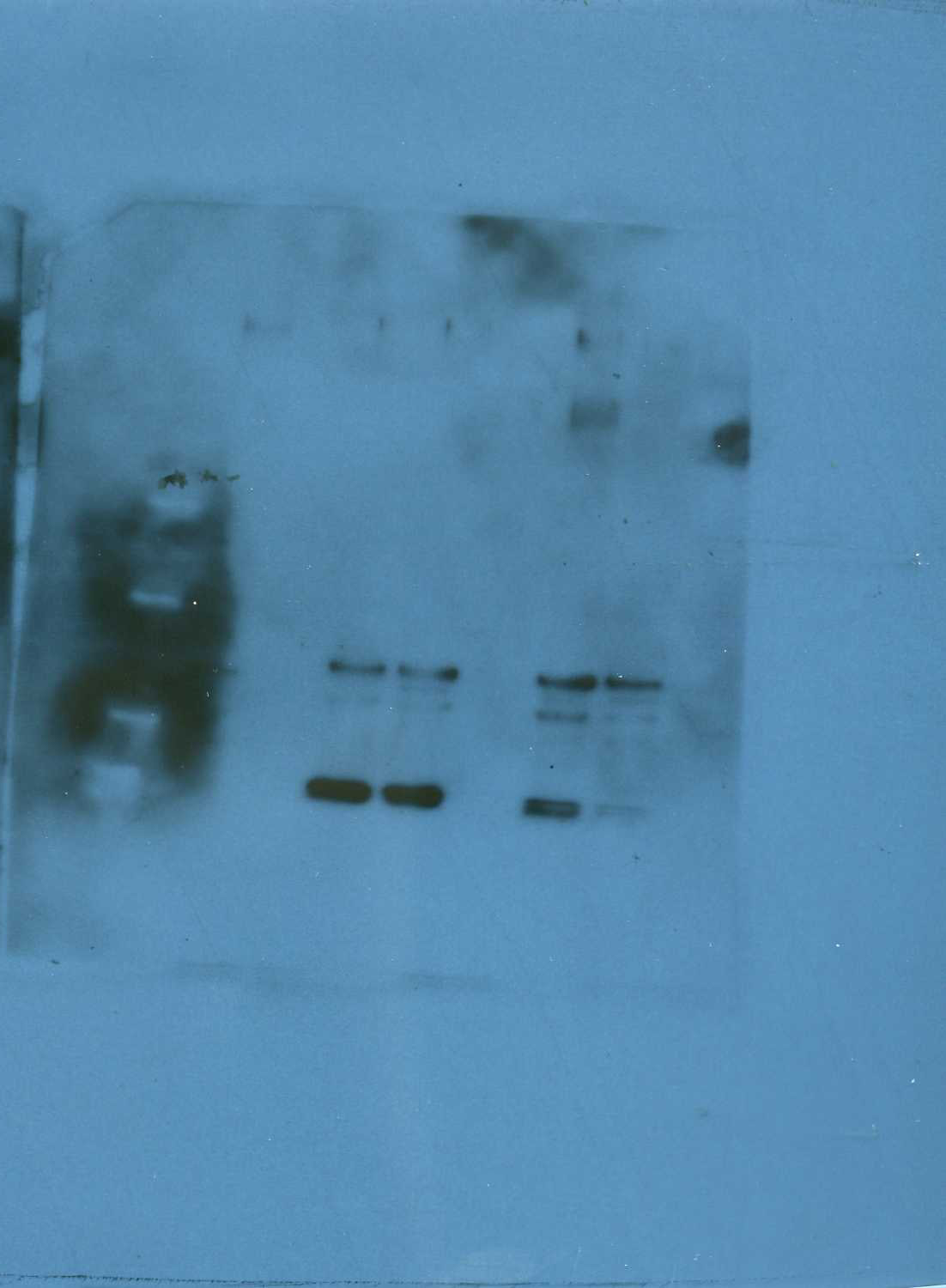

Supplement: Figure 4—figure supplement 2—source data 1. [file elife-86409-fig4-figsupp2-data1.zip › CG17575_raw.jpg]

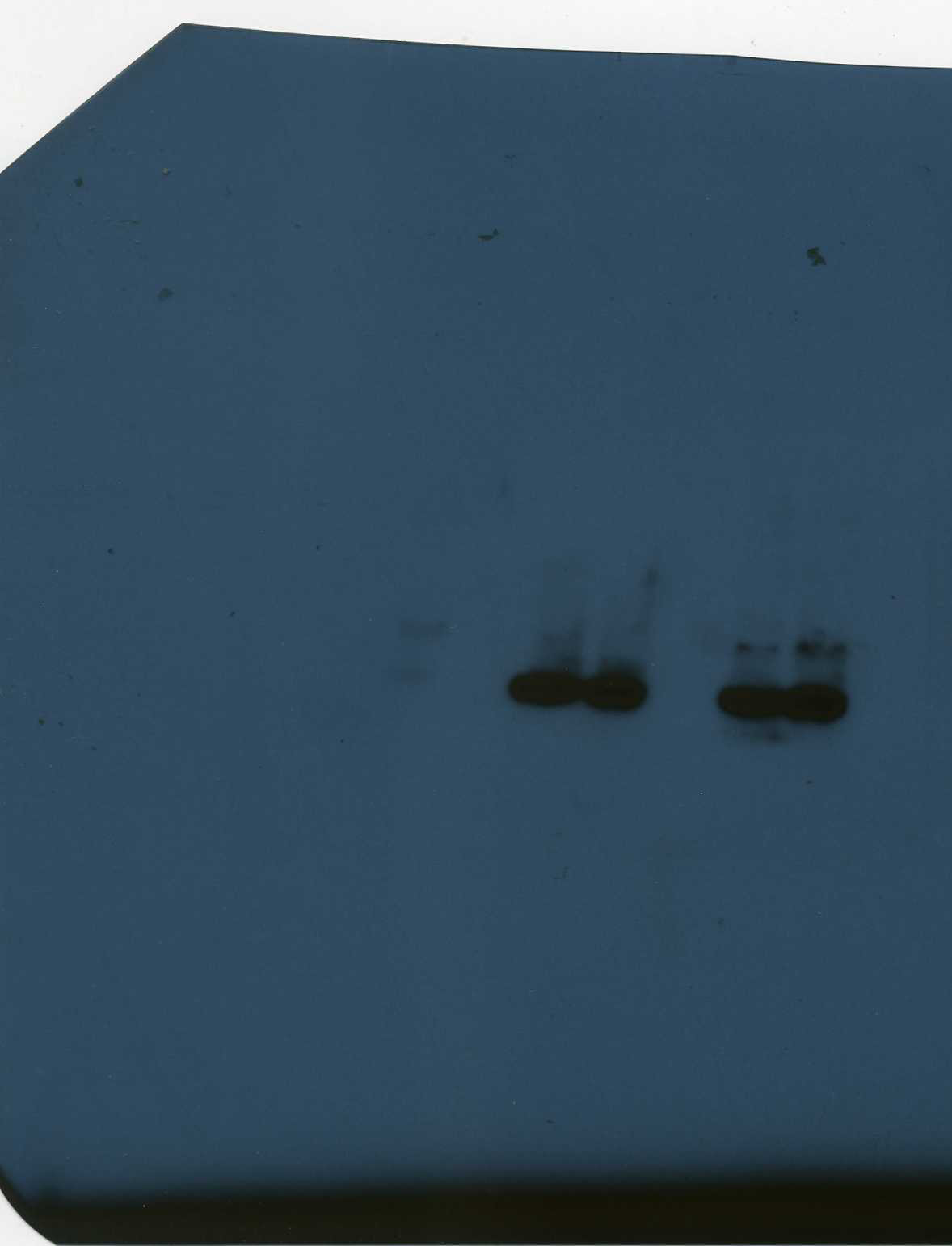

Supplement: Figure 4—figure supplement 2—source data 1. [file elife-86409-fig4-figsupp2-data1.zip › SP_raw.jpg]

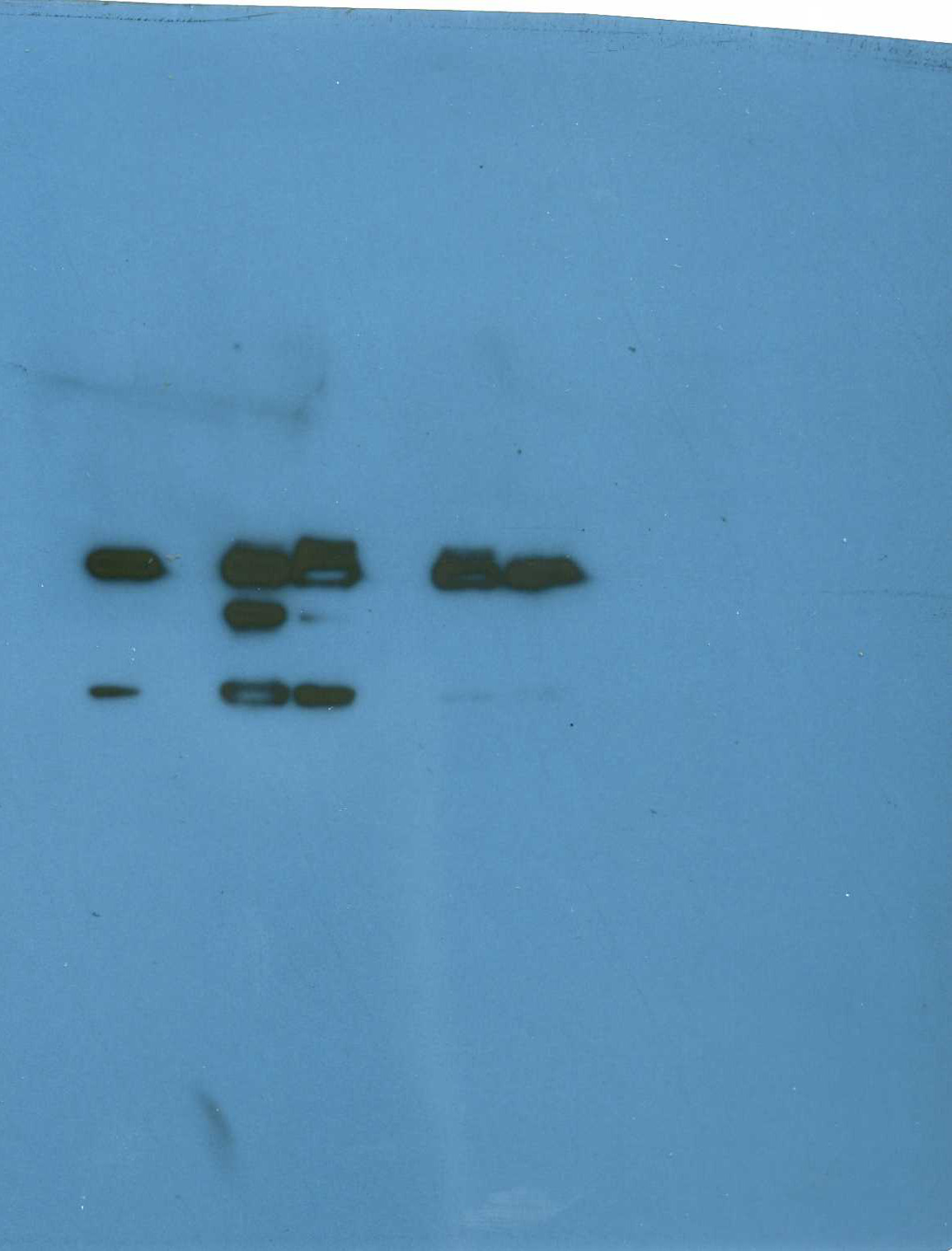

Supplement: Figure 4—figure supplement 2—source data 1. [file elife-86409-fig4-figsupp2-data1.zip › Tubulin_raw.jpg]

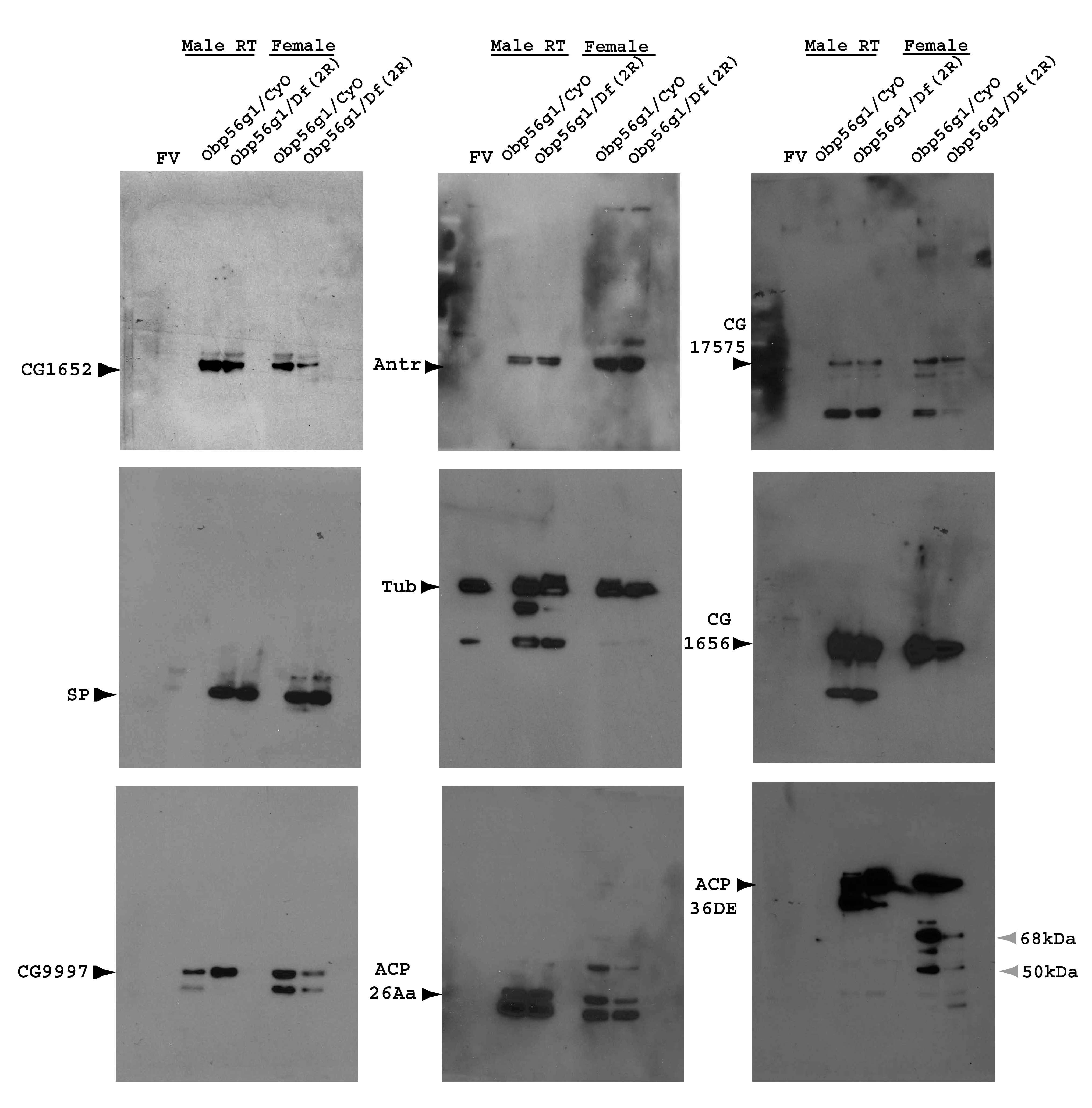

Supplement: Figure 4—figure supplement 2—source data 1. [file elife-86409-fig4-figsupp2-data1.zip › westerns_labeled.jpg]

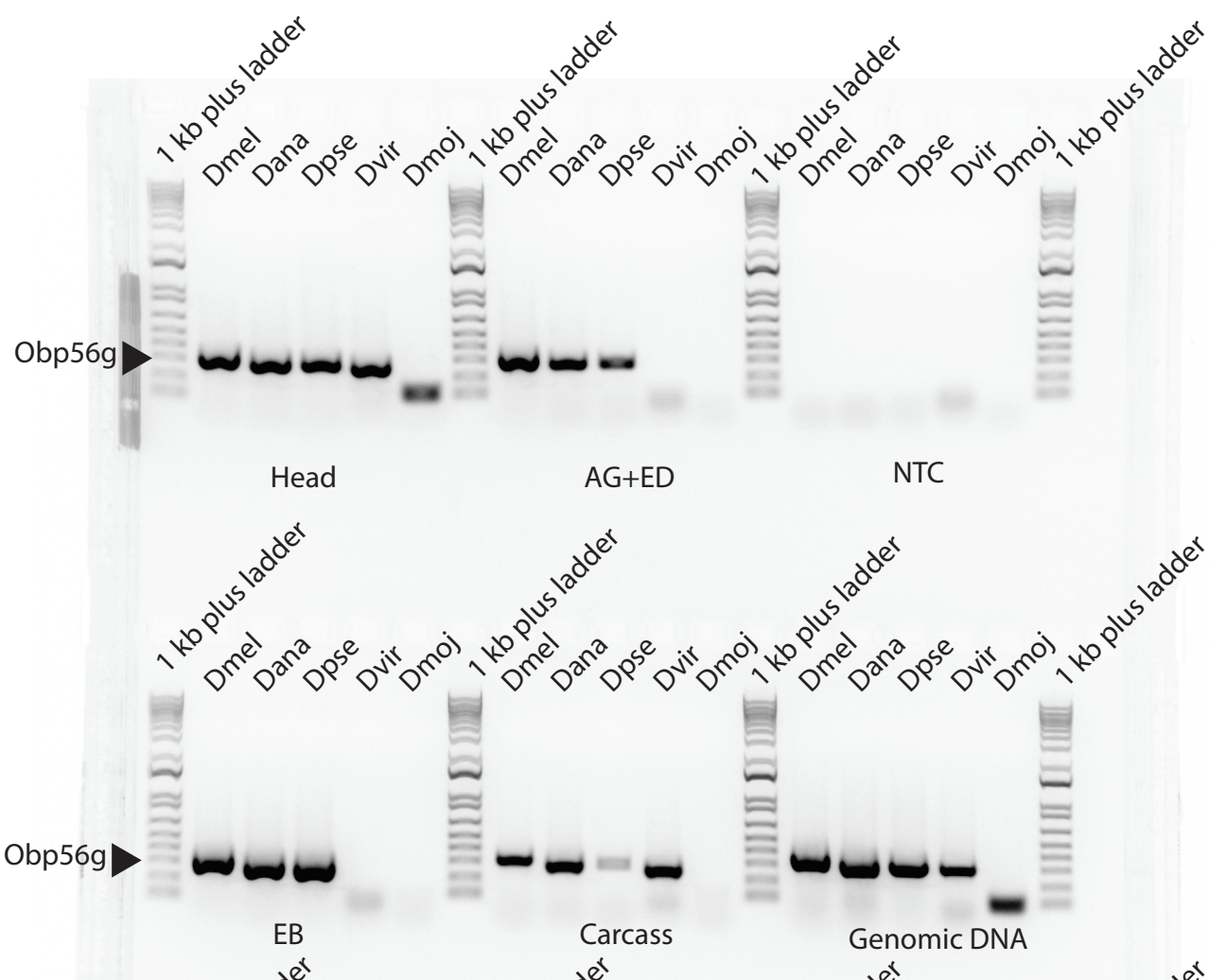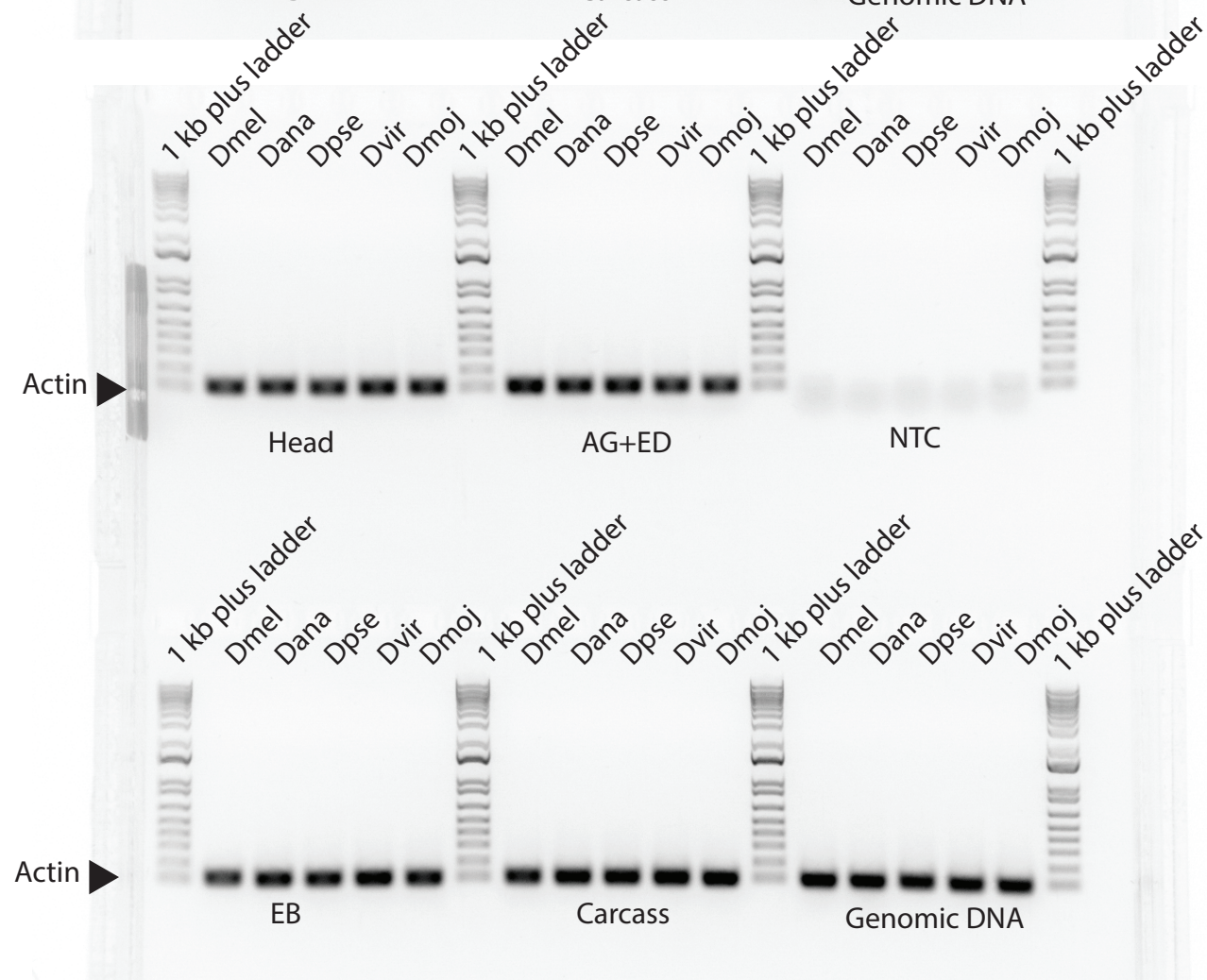

Supplement: Figure 6—figure supplement 1—source data 1. [file elife-86409-fig6-figsupp1-data1.zip › Fig6Supp1_labeled.pdf]

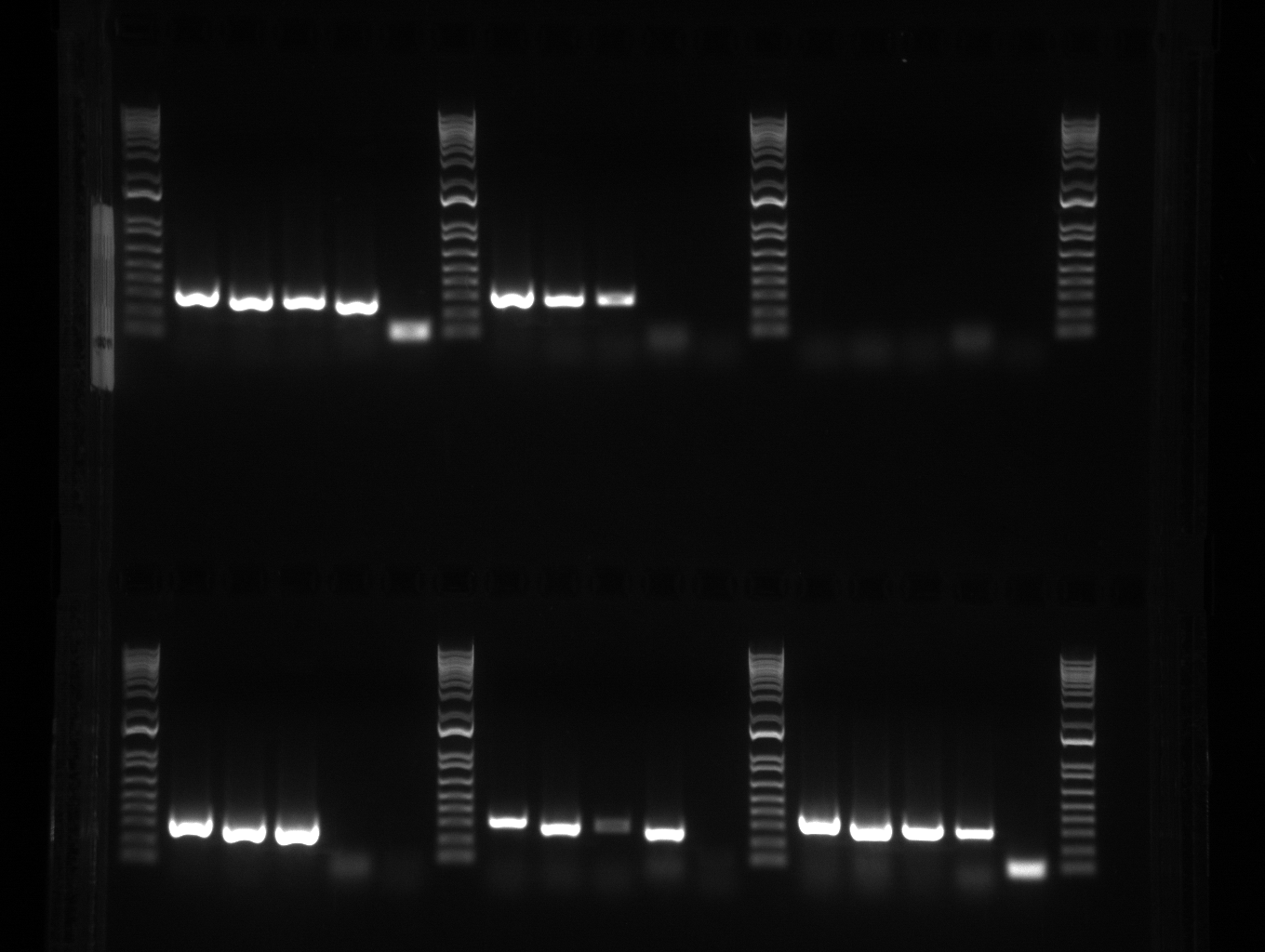

Supplement: Figure 6—figure supplement 1—source data 1. [file elife-86409-fig6-figsupp1-data1.zip › obp56g_raw.tif]

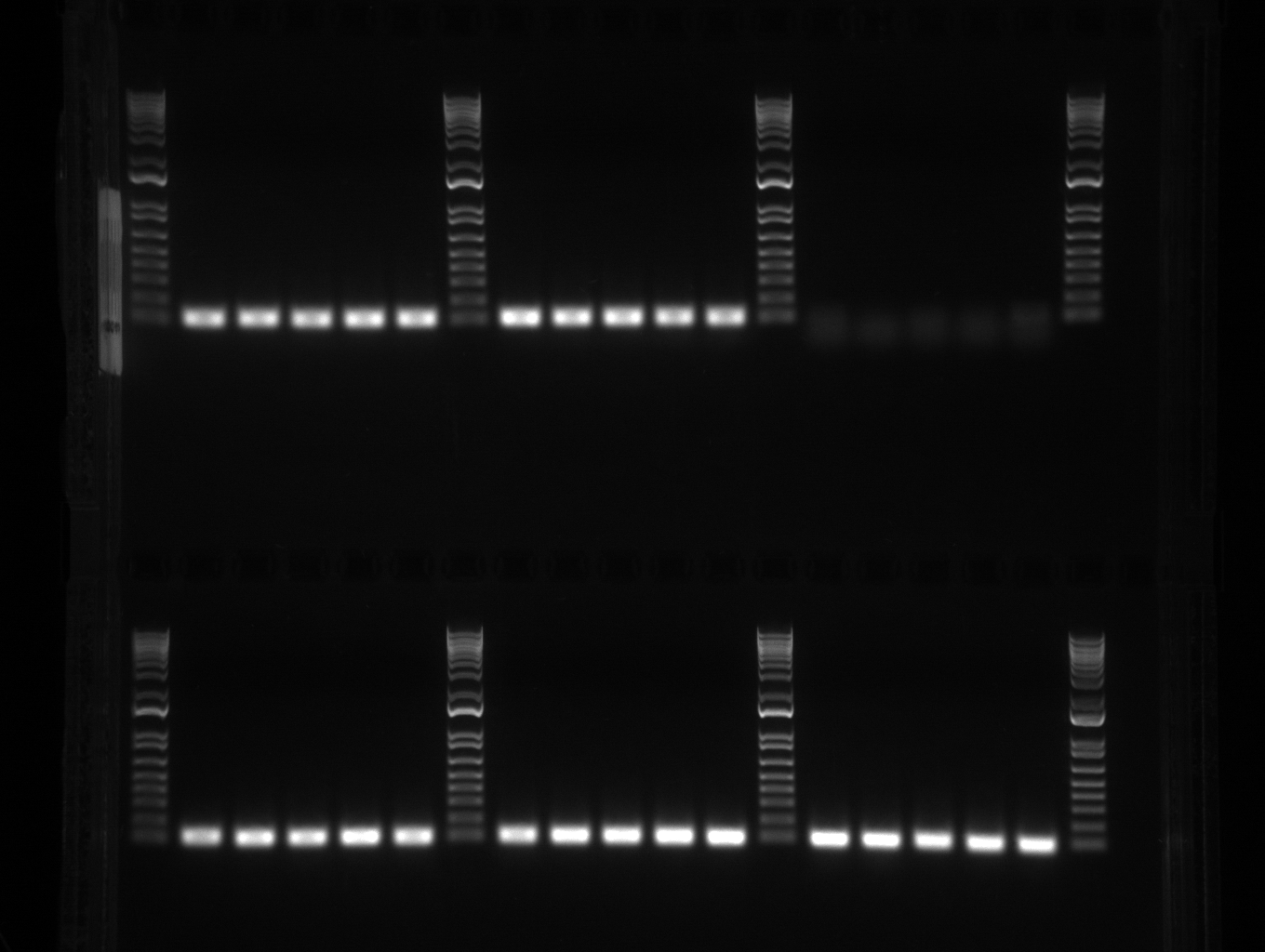

Supplement: Figure 6—figure supplement 1—source data 1. [file elife-86409-fig6-figsupp1-data1.zip › actin_raw.tif]
