# Supplementary material for: The seminal odorant binding protein Obp56g is required for mating plug formation and male fertility in Drosophila melanogaster
Source: eLife. 2023 Dec 21;12:e86409. doi: 10.7554/eLife.86409 (PMC10834028; doi:10.7554/eLife.86409)
Supplement: Supplementary file 1. [file elife-86409-supp1.docx]

| **Gene** | **gRNA sequence (5’->3’)** |
| --- | --- |
| *Obp8a* | 1: GGTGAGGATCGCATGGGCAC  2: GCTGGACAGGATGCAGTTCG  3: ACATGTCCGATGTCATCAAT |
| *Obp22a* | 1: AATTGTAAGCGAGTGTGCCA  2: GAACAATGTTCATAGGAAGA  3: AAAGTGAGGGGGATAGATAG |
| *Obp51a* | 1: TGACAGCTAACAACAGAACC  2: GAACGAATGTGCTAAAAAAC  3: TAAATTCTCGTTTCAAGCAC |
| *Obp56e* | 1: TGAGGCTAAGCAGAGAGCCA  2: CAAGCTATTGCCCTGCGGTC  3: GCCAAGTGTGACTCGACCAA |
| *Obp56f* | 1: AGCCTGCTTGAAACGGCAGC  2: CACTGCTTACTGGAAGTGAA  3: ATGTTTAGAAGTCTAATGCT |
| *Obp56g* | 1: GCAAGCCAACATAGACAGTT  2: CGGTGTCACTCCCCAGGATC  3: CGGATCGTTAAGACCCTAAT |
| *Obp56i* | 1: GGTACAAGCAGGTCCCATTA  2: CGTCATGAGACCGACGACCC  3: CGAAGAACTCGAAATCACAG |
| *ebony* (gRNA sequence from Kane et al., 2017) | GCCACAATTGTCGATCGTCA |

**Table S1 (Supplementary file 1):** gRNA sequences from flyCRISPR’s Optimal Target Finder tool for each *Obp* gene.
