# Supplementary material for: The seminal odorant binding protein Obp56g is required for mating plug formation and male fertility in Drosophila melanogaster
Source: eLife. 2023 Dec 21;12:e86409. doi: 10.7554/eLife.86409 (PMC10834028; doi:10.7554/eLife.86409)
Supplement: Supplementary file 2. [file elife-86409-supp2.docx]

| **Primer** | **Primer Sequence (5’ -> 3’)** |  |
| --- | --- | --- |
| Primer 1F | TTCCCGGCCGATGCAnnnnnnnnnnnnnnnnnnnnGTTTaAGAGCTAtgctgGAAAcag | n: 20nt gRNA 1 |
| Primer 1R | nnnnnnnnnnnnnnnnnnnnTGCACCAGCCGGGAATC | n: 20nt gRNA 2 (RevComp) |
| Primer 2F | nnnnnnnnnnnnnnnnnnnnGTTTaAGAGCTAtgctgGAAAcag | n: 20nt gRNA 2 |
| Primer 2R | nnnnnnnnnnnnnnnnnnnnTGCACCAGCCGGGAATC | n: 20nt gRNA 3 (RevComp) |
| Primer 3F | nnnnnnnnnnnnnnnnnnnnGTTTaAGAGCTAtgctgGAAAcag | n: 20nt gRNA 3 |
| Primer 3R | TTCcagcaTAGCTCTtAAACnnnnnnnnnnnnnnnnnnnnTGCACCAGCCGGGAATC | n: 20nt gRNA 4 (RevComp) |

**Table S2 (Supplementary file 2)**: Primer sequences for cloning gRNAs from Table S1 into pAC-U63-tgRNA-Rev using pMGC as a PCR template (from Poe et al., 2018).
