# Supplementary material for: The seminal odorant binding protein Obp56g is required for mating plug formation and male fertility in Drosophila melanogaster
Source: eLife. 2023 Dec 21;12:e86409. doi: 10.7554/eLife.86409 (PMC10834028; doi:10.7554/eLife.86409)
Supplement: Supplementary file 3. [file elife-86409-supp3.docx]

| **Gene** | **Primer sequence (5’ -> 3’)** | **Purpose of primer pair** |
| --- | --- | --- |
| *Obp8a* | 0F: TCGTAGGTCAGCAGCCCATTAC  0R: TCGCATATGACTTTCAATCCGTGT  1F: CGTGGGAATGATGCGGAGA  1R: CATGGGCAGCATCCTCGAAT | 0: Sequencing CRISPR mutants  1: RT-PCR |
| *Obp22a* | 2F: CCACTTTGTATTGGCAACCGCA  2R: CAGTCCGCCCAACTTTGAGTTT  3F: TGTACTTCTGCTTGGCCTCTC  3R: TTTTGGAAGGATTCTGCACAC | 2: Sequencing CRISPR mutants  3: RT-PCR |
| *Obp51a* | 4F: AGCAATCTCCCTCACGTGATAT  4R: TGCGGCGCTCATGTTTCTTTTA  5F: GGCCTGGTTCTGTTGTTAGC  5R: TCAAGCACTGGAACACCAAG | 4: Sequencing CRISPR mutants  5: RT-PCR |
| *Obp56e* | 6F: ACCTGACAACAAGAAATAACCCGC  6R: CACTAGAGCAAGCGTTCCGTTC  7F: CCCTTGCAGCTCTATCTTTGG  7R: CTTGGTCGAGTCACACTTGG | 6: Sequencing CRISPR mutants  7: RT-PCR |
| *Obp56f* | 8F: GGTAACAGTCCCTGGAAACCGA  8R: GCGCTTTGCCCGGAATAATCTT  9F: TTCATTTTCATCTCTGCTATCTGG  9R: GCCCAATTCACATTTTCCTG | 8: Sequencing CRISPR mutants  9: RT-PCR |
| *Obp56g* | 10F: GTTAGAAACCTTGACAGTGGCA  10R: ATGGGGTAGGCAGTGTATCCCT  11F: AGGGCTACATTCGCATTGAC  11R: ACCTGTCCAAATCCTTTTCG | 10: Sequencing CRISPR mutants  11: RT-PCR |
| *Obp56i* | 12F: ACCTCCATTCGGGTATCTCGAC  12R: GACTGAGTGATGCAAAGCACGT  13F: TGCTGTGCATTATTGTTAGTCG  13R: ACTCGTCATGGGATGTCTCG | 12: Sequencing CRISPR mutants  13: RT-PCR |
| *Actin 5C* | F: AGCGCGGTTACTCTTTCACCAC  R: GTGGCCATCTCCTGCTCAAAGT | RT-PCR control gene |
| *D. ananassae Obp56g* | F: TGACTCTGCTGCTTAGCTGC  R: GATCCTTGTCCACCTGAGCC |  |
| *D. pseudoobscura Obp56g* | F: GGAGCCGGAGACATAAGCAA  R: GCAGGTTTCCTTTCGCATCC |  |
| *D. mojavensis Obp56g* | F: AGAAGCCCGAAATGACCCAG  R: CTCCAGCTTCACCTCACCAG |  |
| *D. virilis Obp56g* | F: GCTGCTTCTCGGCTGTCTAA  R: CCTTAGCTGGCGCATCCTTA |  |

**Table S3 (Supplementary file 3)**: Primer sequences used in this study.
