# Supplementary material for: The seminal odorant binding protein Obp56g is required for mating plug formation and male fertility in Drosophila melanogaster
Source: eLife. 2023 Dec 21;12:e86409. doi: 10.7554/eLife.86409 (PMC10834028; doi:10.7554/eLife.86409)
Supplement: Supplementary file 4. [file elife-86409-supp4.docx]

| **Gene** | **Allele designation** | **Mutant allele description** |
| --- | --- | --- |
| *Obp8a* | *Obp8a^Δ390^* | 390 bp deletion in exon 2 between gRNA 1 + 3 (95% of non-signal peptide sequence) |
| *Obp22a* | *Obp22a^Δ257^* | 257 bp deletion in exon 2 between gRNA 1 + 3 (86% of non-signal peptide sequence) |
| *Obp51a* | *Obp51a^Δ16^* | 16 bp deletion in middle of signal peptide region of exon 1 within gRNA 1 site (predicted frameshift and early stop codon) |
| *Obp56e* | *Obp56e^Δ239^* | 239 bp deletion in exon 2 between gRNA 1 + 3 (69% of non-signal peptide sequence) |
| *Obp56f* | *Obp56f^Δ226^* | 226 bp deletion in exon 2 between gRNA 2 + 3 (67% of non-signal peptide sequence + 13 bp into 3’ UTR) |
| *Obp56g* | *Obp56g^Δ333^* | 333 bp deletion in exon 2 between gRNA 1 + 3 (95% of non-signal peptide sequence + 7 bp into 3’ UTR) |
| *Obp56i* | *Obp56i^Δ359^* | 359 bp deletion in exon 2 between gRNA 1 + 3 (98% of non-signal peptide sequence) |

**Table S4 (Supplementary file 4)**: CRISPR mutant allele summary for each *Obp* gene.
