# Supplementary material for: The seminal odorant binding protein Obp56g is required for mating plug formation and male fertility in Drosophila melanogaster
Source: eLife. 2023 Dec 21;12:e86409. doi: 10.7554/eLife.86409 (PMC10834028; doi:10.7554/eLife.86409)
Supplement: Supplementary file 5. [file elife-86409-supp5.docx]

| **Gene** | **Male genotype** | **% CS females with mating plugs present after copulation** |
| --- | --- | --- |
| *Obp8a* | *Obp8a^Δ390^* | 100% (n=7) |
|  | *Obp8a^WT^* | 100% (n=9) |
| *Obp22a* | *Obp22a^Δ257^* | 100% (n=8) |
|  | *Obp22a^Δ257^ / CyO* | 100% (n=9) |
| *Obp51a* | *Obp51a^Δ16^* | 100% (n=8) |
|  | *Obp51a^Δ16^ / CyO* | 100% (n=8) |
| *Obp56e* | *Obp56e^Δ239^* | 100% (n=8) |
|  | *Obp56e^Δ239^ / CyO* | 100% (n=9) |
| *Obp56f* | *Obp56f^Δ226^* | 90% (n=10) |
|  | *Obp56f^Δ226^ / CyO* | 100% (n=10) |
| *Obp56g* | *Obp56g^Δ333^* | 0% (n=14) |
|  | *Obp56g^Δ333^ / CyO* | 100% (n=11) |
| *Obp56i* | *Obp56i^Δ359^* | 100% (n=10) |
|  | *Obp56i^Δ359^ / CyO* | 100% (n=10) |

**Table S5 (Supplementary file 5)**: Proportion of CS females mated to CRISPR mutant males with morphologically normal mating plugs assessed immediately after the end of mating.
